# Application of Octenidine into Nasal Vestibules Does Not Influence SARS-CoV-2 Detection via PCR or Antigen Test Methods

**DOI:** 10.3390/antibiotics12121724

**Published:** 2023-12-13

**Authors:** Ojan Assadian, Fabiola Sigmund, Daniela Herzog, Karin Riedl, Christoph Klaus

**Affiliations:** 1 Regional Hospital Wiener Neustadt, 2700 Wiener Neustadt, Austria; ojan.assadian@wienerneustadt.lknoe.at (O.A.); fabiola.sigmund@wienerneustadt.lknoe.at (F.S.); daniela.herzog@wienerneustadt.lknoe.at (D.H.); 2 Institute for Skin Integrity and Infection Prevention, University of Huddersfield, Huddersfield HD1 3DH, UK; 3 Schülke & Mayr GmbH, 1070 Vienna, Austria; karin.riedl@schuelke.com

**Keywords:** octenidine, patient decolonization, SARS-CoV-2 detection, nasal decolonization, surgical site infection

## Abstract

The targeted or universal decolonization of patients through octenidine for nasal treatment and antiseptic body wash for 3 to 5 days prior elective surgery has been implemented in several surgical disciplines in order to significantly reduce surgical site infections (SSIs) caused by *Staphylococcus aureus* carriage. However, as most healthcare facilities also screen patients on admission for pilot infection, it is imperative that a prophylactic nasal decolonization procedure not yield a false negative SARS-CoV-2 status in otherwise positive patients. We assessed the effect of a commercially available octenidine-containing nasal gel on two different screening methods—antigen (Ag) detection based on colloidal gold immunochromatography and RT-PCR—in a prospective-type accuracy pilot study in asymptomatic SARS-CoV-2-positive inpatients. All patients still showed a positive test result after using the octenidine-containing nasal gel for about 3 days; therefore, its application did not influence SARS-CoV-2 screening, which is of high clinical relevance. Of note is that Ag detection was less sensitive, regardless of the presence of octenidine. From an infection prevention perspective, these results favor octenidine-based decolonization strategies, even during seasonal SARS-CoV-2 periods. As only asymptomatic patients are considered for elective interventions, screening programs based on RT-PCR technology should be preferred.

## 1. Introduction

Despite the enormous progress in medicine over the past few decades, surgical site infection (SSI) remains a significant complication in surgical disciplines, affecting an estimated 0.5% to 3% of all surgical patients [1,2,3], and can reach even higher numbers in a non-clean surgery or in patients at risk, like obese or diabetic patients. In addition to increased morbidity and mortality, SSIs are associated with additional costs, as patients who contract them are hospitalized approximately 7 to 11 days longer [4]. The appearance of an infection depends on various factors, but a large percentage could be prevented if appropriate strategies are implemented. As SSIs are also caused due to bacteria from the patient’s endogenous flora, most commonly *Staphylococcus aureus*, whole-body decolonization prior elective procedures is known as one simple measure to significantly reduce the risk of development of subsequent infections. Such a bundled intervention typically involves both nasal treatment with an antibiotic (mupirocin) or antiseptic agent (e.g., octenidine) as well as the application of antiseptic skin-cleansing products (e.g., octenidine and chlorhexidine) up to 3–5 days prior to surgery. Scientific evidence to support this recommendation of temporarily reducing or even eliminating potential harmful pathogens is the strongest for cardiothoracic surgeries and prosthetic joint replacement. Of note is that the most commonly used medicinal product worldwide for the pre-surgical patient decolonization of nasal vestibules is based on the antibiotic mupirocin, which is not suspected to be effective against coronavirus or, therefore, to falsify SARS-CoV-2 test results.

However, uncertainties recently arose among healthcare professionals as to whether a decolonization procedure might interfere with SARS-CoV-2 screening programs on admission, as applied antiseptic molecules like octenidine are also known to be effective against several viruses [5].

It has been well investigated that in patients with mild or without typical COVID-19 symptoms, nasopharyngeal RT-PCR tests are superior to antigen (Ag) detection tests regarding sensitivity, as well as specificity [6,7,8]. However, the evaluation of octenidine’s possible influence on both techniques is outstanding so far, and needs to be proven in a clinical setting under practical conditions when an octenidine-containing nasal gel is applied in a 3-day course in (asymptomatic) patients previously confirmed to be SARS-CoV-2-positive.

## 2. Results

As depicted in Table 1, 3 out of 20 (15%) participants were female, and the mean age of the study group was 66.5 ± 13.5 years. Due to the sample size, an allocation regarding sex or age was not performed during patient enrolment; therefore, a further sub-analysis related to test results was not possible. Significantly, we found that nasal decolonization using octenidine did not result in false negative SARS-CoV-2 results when determined via RT-PCR in any of the tested individuals. All patients still showed a positive SARS-CoV-2 test result after 3 days of nasal octenidine application (day 1: Ct [mean] 20.98 ± 5.14; day 3: Ct [mean] 26.36 ± 6.41; Table 1).

A total of 5 out of 20 (25%) and 4 out of 20 (20%) patients showed false negative Ag test results before and after the application of the nasal gel, respectively. Therefore, the sensitivity of the used Ag test was 75% and 80% on day 1 and day 3 in these individuals with no typical symptoms of COVID-19.

What is noteworthy is that in three patients, the Ag test result was negative both times (#8, 14, and 17), although low Ct values were detected prior intervention. Two patients (#4 and 15) showed negative Ag results before using the nasal gel, but were positive after a 3-day-course of octenidine, and only individual #10 tested positive via Ag detection on day 1 but showed a negative result on day 3. SARS-CoV-2 Ag was detectable in all other patients, regardless of the presence or absence of the antiseptic. The Ag-positive percentage agreement was better at day 3 compared to the early stage of infection on day 1 (Table 2).

## 3. Discussion

Numerous recent investigations have confirmed *Staphylococcus aureus* nasal colonization as a key risk factor for the development of SSI in surgical patients [9], increasing the risk of SSI in carriers by 4.5–9.6 times [10,11,12]. In addition, high bacterial load in the nares is associated with a higher likelihood of colonization at other body sites, such as the axillae and groin [13]. When protective skin barriers are disrupted, e.g., during surgery or invasive medical interventions, *Staphylococcus aureus* or other microorganisms can invade or disseminate, causing severe infections such as bacteremia, endocarditis, and pneumoniae. For that reason, the targeted or universal decolonization of patients consisting of antimicrobial agents for nasal treatment and antiseptic body wash several days prior elective surgery has been largely established to significantly reduce SSI rates. Although the World Health Organization (WHO) has recently declared that COVID-19 is no longer a public health emergency of international concern [14], most healthcare facilities actually still screen patients on admission for SARS-CoV-2 in order to avoid a clustering of COVID-19 patients, particularly in high-risk areas. Moreover, depending on new mutations of the virus, further epidemic scenarios are, of course, likely. Hence, it is imperative that a prophylactic nasal decolonization procedure not yield false negative SARS-CoV-2 status in otherwise positive patients.

The main objective of the present clinical pilot study was to prospectively collect human nasal specimens to determine whether a 3-day-course application of an antimicrobial nasal gel might influence the coronavirus screening results performed with a lateral flow Ag test and a RT-PCR assay for the detection of SARS-CoV-2. Usually, the antibiotic mupirocin for nasal cavity is widely used for SSI prevention, which is unsuspected to be effective against the virus, but to the best of our knowledge, the possible interference of the also widely used antiseptic molecule octenidine has not been investigated so far. Due to global concern about antibiotic resistance and because of improved antibiotic stewardship, octenidine is increasingly used in patient decolonization protocols in Europe, Australia, and some Asian countries [15,16,17,18,19,20]. In contrast to the specific mode of action of mupirocin against Staphylococcus species, the antiseptic immediately targets and destroys the structure of lipid membranes [21,22] and shows a much broader spectrum of antimicrobial efficacy, including against enveloped viruses [5].

Among 20 paired samples from COVID-19-asymptomatic, SARS-CoV-2–positive individuals, overall Ag test sensitivity was 77.5% (31/40): 75.0% (15/20) before intervention and 80.0% (16/20) 3 days later, compared to the current diagnostic gold standard, RT-PCR. This rate was non-significantly different on day 3; therefore, the here-performed intervention by using an antiseptic did not influence test results based on Ag or RT-PCR. The relatively low rate in symptomless people was expected, and is in line with findings from previously performed clinical trials [23,24], which have been obtained for different commercially available Ag test systems. Marx et al. [25] investigated the difference in SARS-CoV-2 detection between saliva and anterior nasal specimens compared with nasopharyngeal samples, and found an overall sensitivity of 85% vs. 80% (saliva vs. anterior) and an even more pronounced effect among symptomatic participants than among those without symptoms (94% vs. 29% for saliva; 87% vs. 50% for anterior nasal samples) when using RT-PCR. Hence, it was clear that anterior nasal swabs are inferior to nasopharyngeal specimens, even when using the more sensitive nucleic acid amplification technology (NAAT). False negative results might lead to failures in infection control and prevention practices; therefore, low sensitivity supports the strategy of PCR. As only symptomless patients are considered for elective interventions, screening programs based on NAAT should be always preferred [26].

Nevertheless, both methods are still widely used in daily clinical practice, and we wanted to investigate a possible interference with a frequently used octenidine-based nasal gel. To provide clarity to users of both options, we included the rapid Ag detection test (for anterior samples), as well as a RT-PCR (for nasopharyngeal samples) in the present study. This design was chosen because octenidine is applied into the nasal vestibules; therefore, it was of high interest as to whether its impact is was likely to be more pronounced on anterior swabs than on nasopharyngeal specimens used for SARS-CoV-2 screening. In fact, and of high clinical importance, use of the nasal gel over a period of 3 days did not affect either of the two coronavirus test methods, since patients still showed a positive result.

Of note is that in our setting, octenidine was neither intended to be used for SARS-CoV-2 therapy nor to reduce the amount of viral load in respiratory droplets and aerosols exhaled by infected individuals. Virus particles originating from the lungs constantly re-contaminate nasal vestibules through continuous exhalation. As the used Ag test simply detects viral protein in anterior samples, regardless of whether it derives from infectious or already inactivated coronavirus, the obtained positive test result does not automatically provide information on the clinical effectiveness of the octenidine-based nasal gel on SARS-CoV-2. Whether the verified viral material in the anterior samples was still infectious was not separately analyzed in the present study, but definitely represents an important topic for further research.

The clinical pilot project included only a low number of a total of 20 participants and has another limitation. According to manufacturers’ instruction for use, a nasopharyngeal swab was taken for RT-PCR, whereas an anterior nasal swab was performed for the Ag test. Hence, we did not compare exactly the same source of patient material. However, since the nasal gel is supposed to be used in nasal vestibules, we were aware of this disparity and wanted to investigate whether the nasal gel was likely to interfere with this test method when swabs were taken anteriorly. Additionally, we used only one test system per method, as mentioned in the Materials and Methods section, both of which were already implemented and available in our healthcare facility. We found a better Ag-positive percentage agreement at day 3—and after the application of the nasal gel—compared to the early stage of infection on day 1; however, our sample size was too small to show statistical significance correlated to clustered Ct-values.

## 4. Materials and Methods

### 4.1. Study Design

For this prospective-type accuracy pilot study, previously confirmed SARS-CoV-2-positive inpatients (RT-PCR Ct values < 40) older than 18 years with no or mild COVID-19 symptoms were included. Exclusion criteria were defined as body temperature ≥ 38 °C, reported respiratory distress, shortness of breath, cough, flu-like symptoms, loss of taste, vomiting, diarrhea, or headache.

After giving written informed consent, a total of 20 patients were asked to apply a pea-sized amount of octenidine-based nasal gel (octenisan^®^ md nasal gel, Schülke & Mayr GmbH, Vienna, Austria) into both nasal vestibules twice daily for 3 consecutive days, according to the manufacturer’s manual. All study participants received comprehensive verbal instructions by the study staff, as well as written information on the application procedure. Compliance was checked on separate patient data sheets. Swabs were taken by healthcare professionals immediately prior to the first application of the nasal gel (day 1), as well as at the end of the intervention (day 3). This period is recommended according to the instructions for use of the nasal gel, and was therefore chosen to investigate its possible influence on SARS-CoV-2 screening methods. Nasal swabs for Ag analysis were collected from both nostrils by gently twisting and pushing the swab against the inner wall of the nasal vestibule according to manufacturer recommendation. Nasopharyngeal swabs for RT-PCR analysis were taken by slightly tilting the patient’s head back and inserting the swab through one nostril parallel to the palate while rotating until resistance was encountered or the distance was equivalent to that from the ear to the nostril of the patient, indicating contact with the nasopharynx [27].

### 4.2. RT-PCR Test

For RT-PCR testing, nasopharyngeal specimens were analyzed using Xpert^®^ Xpress CoV plus kits in GeneXpert^®^ systems (both Cepheid Inc., Whitesboro, NY, USA) according the manufacturer’s instructions. Viral load was determined in a semiquantitative manner, expressed by the number of PCR cycles needed to amplify RNA to a detectable level, termed cycle threshold (Ct). Ct values < 40 were used to define a positive RT-PCR result.

### 4.3. Antigen (Ag) Rapid Diagnostic Test

Ag tests of nasal specimens were performed by using the SARS-CoV-2 Antigen Rapid Test Kit (Shenzehn Lvshiyuan Biotechnology Co. Ltd., Shenzhen, China). This commercially available lateral flow assay is based on colloidal gold immunochromatography using the double-antibody sandwich method. A visible red test and control line indicated a positive result. As numerous CE-marked Ag rapid diagnostic tests are offered in Europe, showing huge differences in sensitivity, the SARS-CoV-2 Antigen Rapid Test Kit was chosen because its performance was proven to be one of the highest [6].

## 5. Conclusions

The application of an octenidine-based nasal gel, when used for the pre-surgical decontamination of patients, did not influence SARS-CoV-2 screening on admission to a tertiary care hospital, and is thus an appropriate measure for SSI prevention, even during seasonal SARS-CoV-2 epidemics or pandemics. This finding is of relevance to avoid nosocomial clusters in healthcare facilities through unidentified SARS-CoV-2-positive patients due to false negative results.

## Figures and Tables

**Table 1:** SARS-CoV-2 RT-PCR (Ct value) and Ag detection (+ positive, − negative) test results for indicated patients (m = male, f = female, age in years) immediately before applying the octenidine-based nasal gel on day 1 (d1) and after the intervention on day 3 (d3).

Patient #	d1		d3	
	RT-PCR (Ct)	Ag	RT-PCR (Ct)	Ag
1 (f, 53)	33.8	+	25.6	+
2 (m, 80)	17.2	+	21.1	+
3 (m, 77)	18.3	+	32.8	+
4 (m, 80)	28.2	−	36.9	+
5 (m, 29)	14.2	+	26.9	+
6 (m, 79)	13.9	+	23.1	+
7 (m, 62)	22.0	+	26.1	+
8 (m, 87)	17.7	−	27.1	−
9 (m, 73)	27.3	+	12.3	+
10 (m, 72)	22.2	+	30.2	−
11 (m, 82)	28.5	+	20.1	+
12 (m, 85)	22.7	+	19.3	+
13 (m, 63)	20.9	+	29.7	+
14 (f, 61)	19.1	−	34.1	−
15 (f, 53)	14.0	−	17.3	+
16 (m, 85)	23.2	+	27.9	+
17 (m, 73)	18.1	−	39.4	−
18 (m, 79)	21.1	+	24.9	+
19 (m, 89)	17.1	+	26.1	+
20 (m, 50)	20.0	+	26.3	+

**Table 2:** Antigen-positive percentage agreement (PPA) stratified by PCR-Ct values shown for day 1 (d1) and day 3 (d3), respectively. n.c. = not calculable.

RT-PCR (Ct)	>10–15	>15–20	>20–25	>25–30	>30–35	>35
	d1 (d3)	d1 (d3)	d1 (d3)	d1 (d3)	d1 (d3)	d1 (d3)
Ag-positive	2 (1)	4 (2)	6 (4)	2 (7)	1 (1)	0 (1)
Ag-negative	1 (0)	3 (0)	0 (0)	1 (1)	0 (2)	0 (1)
PPA	66.7% (100%)	57.1% (100%)	100% (100%)	66.7% (87.5%)	100% (33.3%)	n.c. (50%)

## Data Availability

Data are contained within the article.
